# Supplementary material for: The Levels of Circulating MicroRNAs at 6-Hour Cardiac Arrest Can Predict 6-Month Poor Neurological Outcome
Source: Diagnostics (Basel). 2021 Oct 15;11(10):1905. doi: 10.3390/diagnostics11101905 (PMC8534364; doi:10.3390/diagnostics11101905)
Supplement: Supplementary file 1 [file diagnostics-11-01905-s001.zip › diagnostics-1373956-supplementary.pdf]

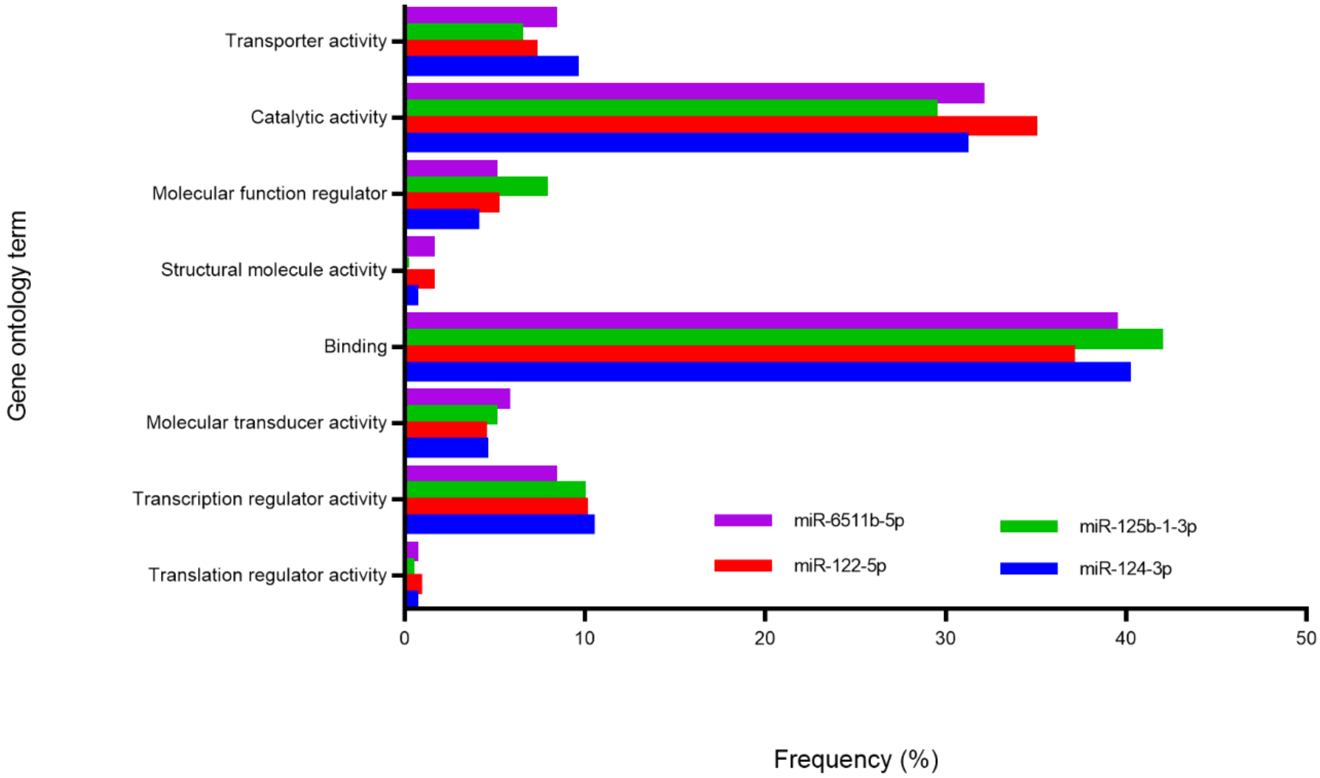

Figure S1. Gene ontology analysis of miR-6511b-5p, -125b-1-3p, -122-5p, and -124-3p.

Table S1. Baseline characteristics of the discovery cohort.

	Healthy controls (n=4)	Cardiac arrest survivors (n=11)	p-value
Male	2 (50.0)	7 (63.6)	1.000
Age, year, median (IQR)	57.0 (51.8–59.3)	60.0 (55.0–70.0)	0.395
Comorbidities			
Hypertension	1 (25.0)	3 (27.3))	1.000
Diabetes mellitus	1 (25.0)	4 (36.4)	1.000
Ischaemic heart disease	0 (0.0)	1 (9.1)	1.000
Chronic heart failure	0 (0.0)	0 (0.0)	NA
Stroke	0 (0.0)	0 (0.0)	NA
Chronic obstructive pulmonary disease	0 (0.0)	2 (18.2)	1.000
Chronic renal disease	0 (5.0)	1 (9.1)	1.000
Liver failure	0 (0.0)	0 (0.0)	NA
Malignancy	0 (0.0)	0 (0.0)	NA

Data are presented as n (%) for categorical variables unless otherwise indicated.

IQR, interquartile range.

Table S2. Summary of small RNA sequencing in a small cohort.

Sample ID	Group	Total reads	Passing filters (%)	Aligned reads (%)	Precursor miRNA reads	Mature miRNA reads	Known precursor with ≥ 5x coverage	No. of known miRNAs	No. of novel miRNAs
S0001	Poor	11858483	93.3	87.58	8007	5295476	546	682	276
S0002	Good	12467717	93.21	35.63	41930	3547980	667	542	254
S0003	Control	8226664	98.83	76.96	4204	9525344	266	490	170
S0004	Control	8177899	98.04	65.36	30817	12021794	353	420	179
S0005	Good	11424524	99.38	20.34	4016	2188300	564	413	130
S0006	Good	11690165	99.71	43.17	2589	5936130	505	401	143
S0007	Poor	17541976	99.36	82.24	8103	19966958	798	550	401
S0008	Poor	18769268	97.42	66.52	10097	11981713	1016	787	226
S0009	Poor	12380592	99.32	77.26	4753	10700210	496	366	95
S0010	Good	21698276	99.28	70.82	6629	19190859	478	372	213
S0011	Poor	11721780	97.96	66.33		6590901	540	398	199
S0012	Poor	11382580	98.63	71.13	4209	5387947	674	468	334
S0013	Good	11084020	98.55	51.05	2501	9068805	646	371	146
S0014	Control	10537839	98.44	76.93	9309	7445048	901	248	120
S0015	Control	11663439	98.80	81.45	7376	4159462	484	281	104

Table S3. Comparisons of miRNA expression levels between patients with good outcome and poor outcome.

	Good outcome (n=20)	Poor outcome (n=34)	p-value
miR-6511b-5p	0.019 (0.092–0.048)	0.128 (0.046–0.523)	<0.001
miR-125b-1-3p	0.027 (0.010–0.051)	0.202 (0.035–0.665)	<0.001
miR-122-5p	0.003 (0.000–0.008)	0.038 (0.004–0.130)	0.001
miR-124-3p	0.005 (0.001–0.008)	0.018 (0.008–0.340)	0.001
miR-511-5p*	0.018 (0.006–0.039)	0.147 (0.044–0.873)	0.001
miR-519a-3p*	0.000 (0.000–0.520)	0.134 (0.161–0.393)	0.003
miR-18b-3p	0.061 (0.026–0.009)	0.206 (0.071–0.514)	0.025
miR-3180-3p*	0.001 (0.000–0.003)	0.005 (0.001–0.235)	0.036
miR-24-2-5p*	0.069 (0.020–0.383)	0.255 (0.048–0.826)	0.114
miR-590-3p*	0.105 (0.017–0.785)	0.099 (0.022–0.718)	0.907

*n=47 (good outcome, 14; poor outcome, 33).

Table S4. miRNA expression levels according to the hemodynamic state.

	Shock on admission (+) (n=19)	Shock on admission (-) (n=35)	p-value	Need for vasopressor (+) (n=31)	Need for vasopressor (-) (n=23)	p-value
miR-6511b-5p	0136 (0.053–0.464)	0.043 (0.011–0.109)	0.020	0.116 (0.023-0.369)	0.043 (0.011-0.075)	0.027
miR-125b-1-3p	0.255 (0.059–1.291)	0.035 (0.020–0127)	0.005	0.150 (0.032-0.619)	0.035 (0.015-0.095)	0.003
miR-122-5p	0.045 (0.009–0.154)	0.005 (0.001–0.020)	0.008	0.038 (0.004-0.122)	0.004 (0.001-0.008)	0.002
miR-124-3p	0.019 (0.008–0.115)	0.008 (0.001–0.017)	0.069	0.012 (0.001-0.334)	0.008 (0.004-0.015)	0.231

Data are presented as the median (interquartile range)

Table S5. The correlation coefficient between miRNAs and other laboratory values.

	miR-6511b-5p	miR-125b-1-3p	miR-122-5p	miR-124-3p
Troponin T	0.594, p < 0.001	0.613, p < 0.001	0.277, p=0.043	0.469, p < 0.001
N-terminal pro-brain natriuretic peptide	0.424, p=0.001	0.399, p=0.003	0.219, p=0.112	0.221, p=108
Bilirubin	0.041, p=0.770	0.124, p=0.370	0.352, p=0.009	-0.063 p = 0.652
